# Early findings from the integration of hypertension care into differentiated service delivery models for HIV in Uganda: a mixed‐method study

**DOI:** 10.1002/jia2.26499

**Published:** 2025-07-07

**Authors:** John Baptist Kiggundu, Fred C. Semitala, Chelsea Faith Lipoto, Lilian Giibwa, Robert Twine, Savio Mwaka, Florence Ayebare, Christine Kiwala, Evelyn N. Magambo, Gerald Mutungi, Isaac Ssinabulya, Donna Spiegelman, James Kayima, Martin Muddu, Jeremy I. Schwartz, Anne R. Katahoire, Chris T. Longenecker

**Affiliations:** ^1^ Infectious Diseases Research Collaboration Kampala Uganda; ^2^ Department of Internal Medicine Makerere University Kampala Uganda; ^3^ Makerere University Joint AIDS Program Kampala Uganda; ^4^ Department of Non‐Communicable Diseases Ministry of Health Kampala Uganda; ^5^ Uganda Heart Institute Kampala Uganda; ^6^ Uganda Initiative for Integrated Management of Non‐Communicable Diseases Kampala Uganda; ^7^ Department of Biostatistics and Center for Methods on Implementation and Prevention Science (CMIPS) Yale School of Public Health New Haven Connecticut USA; ^8^ Section of General Internal Medicine Yale School of Medicine New Haven Connecticut USA; ^9^ Child Health and Development Centre Department of Medicine Makerere University Kampala Uganda; ^10^ Division of Cardiology Department of Global Health University of Washington Seattle Washington USA

**Keywords:** differentiated service delivery, HIV, hypertension, implementation, integrated care, people living with HIV

## Abstract

**Introduction:**

Uganda's national guidelines recommend integrated HIV and hypertension care; however, integration of hypertension care into HIV differentiated service delivery (DSD) models has not been extensively described. We aimed to describe trends in DSD models for people living with HIV (PLHIV) with hypertension and to qualitatively describe the experiences of healthcare providers (HCPs) and PLHIV with hypertension after implementing integrated care.

**Methods:**

We conducted a parallel convergent mixed methods study nested in an ongoing stepped wedge cluster randomised trial in Kampala and Wakiso districts. Quantitative data (age, sex, blood pressure, DSD model, medication prescriptions) were collected from routine medical records at eight clinics implementing the enhanced care package between March 2023 and July 2024. Additionally, structured interviews were conducted at two clinics with HCPs (*n* = 6, 3 per clinic) and PLHIV with hypertension (*n* = 8, 4 per clinic). Our quantitative outcome variable was enrolment in intensive DSD models (facility‐based individual and group models) versus other DSDs. A generalised estimation equation was used to account for within clinic correlation and repeated measures within participants over time. Inductive thematic analysis was applied to the qualitative data using the Consolidated Framework for Implementation Research.

**Results:**

Overall, 3164 PLHIV with hypertension accessed care at the eight clinics. Median age was 46 years (IQR 38–56); more than two‐thirds were female. There was considerable heterogeneity across clinics in the use of DSD models during the study period. Overall, use of intensive models increased over time (OR 1.127 [1.059−1.199] per month). However, two clinics showed significant time interaction effects (Wald test χ^2^ (7) = 69.94, *p* < 0.001), with a decrease in the intensive models over time. HCPs and PLHIV observed that integrating hypertension care was easily adaptable in some models, while more challenging in others. The availability of resources and synchronisation of HIV and hypertension visits facilitated the integration of hypertension care within the HIV DSD models.

**Conclusions:**

The integration of hypertension management into HIV DSD models is both feasible and adaptable; however, it requires transitioning PLHIV between various models based on clinical needs. To facilitate this process, comprehensive client education by the HCPs is necessary.

**Clinical Trial Number:**

clinicaltrials.gov # NCT05609513

## 1 INTRODUCTION

Differentiated service delivery (DSD) models of care are defined as “client‐centered approaches that simplify and adapt HIV services across the cascade, in ways that both serve the needs of people living with HIV (PLHIV) better and reduce unnecessary burdens on the health system” [1]. Uganda adopted and began to scale up DSD models in 2017 [2]. By 2023, approximately 1.4 million adult PLHIV were receiving care through one of the six DSD models, experiencing well‐documented benefits such as fewer clinic visits, reduced waiting time and lower transportation costs [1, 3, 4]. From the health system's perspective, healthcare providers (HCPs) experience decreased client load and reduced workload, while the system provides more efficient care at lower cost [4, 5].

Like many other sub‐Saharan African countries, Uganda faces a significant burden of both infectious and non‐communicable diseases (NCDs) [6, 7]. Up to 20–32% of adult PLHIV have hypertension, a leading preventable risk factor for cardiovascular diseases (CVDs) [8, 9, 10, 11]. PLHIV with hypertension have a higher risk of CVD and all‐cause mortality compared to people without HIV [12, 13]. For these reasons, inclusive, person‐centred hypertension treatment should be a top priority within HIV DSD models.

With the increasing burden of hypertension among PLHIV, some countries in Africa including Uganda have recommended the integration of hypertension in HIV care across all DSD models [14]. For example, the Uganda guidelines for HIV treatment recommend that PLHIV with controlled hypertension receive care through both less intensive and intensive models, while those with uncontrolled hypertension should receive care through intensive models, such as facility‐based individual management (FBIM) and facility‐based group (FBG) care, or HCP‐managed less intensive models, including community drug distribution points (CDDPs) and fast track drug refill (FTDR) models [15, 16].

Although prior implementation and programmatic reports have demonstrated improved hypertension cascade metrics following the integration of HIV hypertension care, most of these studies have focused on integrating hypertension in intensive models [17, 18]. Given that nearly 80% of PLHIV in Uganda receive care through less intensive models [19], PLHIV with hypertension are likely to receive suboptimal hypertension care if integration is not expanded to all models. Several groups have proposed expanding DSD models to offer more comprehensive and person‐centred care, including hypertension services [20, 21, 22, 23]. However, there is limited evidence to show how the implementation of hypertension services in less intensive models can be done. In addition, there is little information detailing the experiences of PLHIV receiving HIV and hypertension care through these models.

The Strengthening Blood Pressure Care and Treatment Cascade for Ugandans Living with HIV—ImpLEmentation Strategies to SAve Lives (PULESA‐Uganda) study is an implementation trial aimed at improving hypertension care cascades among PLHIV [24, 25]. The ongoing PULESA‐Uganda trial leverages existing HIV platforms to expand care for hypertension across public and private not‐for‐profit clinics. In this paper, we document trends in the utilisation of the various DSD models for PLHIV with hypertension following the integration of HIV hypertension care at these clinics. We also explore the experiences of HCPs in delivering, and PLHIV with hypertension in receiving, hypertension care within DSD models using the updated Consolidated Framework for Implementation Research (CFIR) [26].

## 2 METHODS

### 2.1 Study design

We conducted a parallel convergent mixed methods study nested in PULESA‐Uganda, a stepped wedge cluster randomised trial evaluating two strategies for integrating HIV hypertension care in 16 HIV clinics in Wakiso and Kampala districts [25]. Eight clinics were randomly assigned to “Hypertension PLUS” or “Hypertension BASIC.”

Clinics assigned to “Hypertension BASIC” receive a consistent supply of guideline‐recommended anti‐hypertensive medications (amlodipine 10 mg, valsartan 160 mg and hydrochlorothiazide 25 mg), basic training on hypertension treatment and blood pressure (BP) devices *(Welch Allyn ProBP 2000 digital)*. In addition to the components of “Hypertension BASIC,” clinics randomised to the “Hypertension PLUS” arm of the study receive: (1) training of HCPs on hypertension care; (2) quarterly performance audit and feedback; (3) remote patient monitoring of BP in the community; and (4) integration of HIV hypertension care in all existing HIV DSD models while aligning both HIV hypertension services and allowing multi‐month dispensing (MMD) for both anti‐retroviral therapy (ART) and anti‐hypertensive medications (Supporting information File S1).

### 2.2 Study sites

Although 16 clinics are participating in the trial, this paper presents initial findings from the eight clinics assigned to the “Hypertension PLUS” intervention who are more intentionally working to integrate hypertension care into existing DSD models as one of the package components. These clinics were assigned random unique identifiers (C12, C01, C14, C08, C03, C10, C05, C15) and are subsequently referred to by these numbers in our manuscript (Supporting information File S2).

All the HIV clinics are supported by a local implementing partner with funding from the US President's Emergency Fund for AIDS Relief to provide HIV care and treatment services. PLHIV receive care and treatment services under the six DSD models; FBIM, FBG, Fast Track Drug Refill, Community Client‐Led ART Delivery, Outreach HCP‐led Community Drug Distribution Point (CDDP‐outreach) and Community Retail Pharmacy Drug Distribution Point (CRPDDP) [15] (Supporting information File S3).

### 2.3 Participant selection

For the quantitative component, we gathered routine clinical data from PLHIV aged 18 years and above who received HIV and hypertension care at the “Hypertension PLUS” clinics. Using the Uganda guidelines, participants were classified as having hypertension if they had at least two BP readings ≥ 140/90 mmHg from separate clinic visits a week apart or were on anti‐hypertensive treatment [15]. Although we did not have a predetermined sample size, the power calculations for the primary outcomes were based on an estimated sample size of 5000 PLHIV with hypertension across all 16 clinics.

We purposively selected a sub‐sample of eight PLHIV with hypertension (*n* = 4 per clinic) aged 18 years and above and provided informed consent to participate in in‐depth interviews (IDIs). We excluded those with cognitive impairment or using anti‐hypertensive medications for purposes other than treating hypertension (e.g. systolic heart failure). Our purposive approach to sampling sought to balance the representation of natal sex as well as participants diagnosed with hypertension either prior to or during the intervention rollout.

For key informant interviews (KIIs), we purposively selected six HCPs (*n* = 3 per clinic) who provided integrated HIV and hypertension care. We excluded HCPs not engaged in direct client care or planning to leave the clinics during the study period.

### 2.4 Data collection

#### 2.4.1 Quantitative data

From March 2023 to July 2024, trained research assistants extracted data from paper‐based medical records of adult PLHIV at each clinic on every clinic visit during the implementation phase. Due to the stepped‐wedge design, follow‐up time ranges from 2 to 16 months (Supporting information File S4). We collected age, sex, BP measurements, chart diagnosis of hypertension, anti‐hypertensive medications, DSD model and place of prescription. These data were entered in a pre‐designed instrument developed in Research electronic data capture [27, 28]. The study data manager conducted weekly data quality checks to ensure that all metrics were correctly entered.

#### 2.4.2 Qualitative data

We conducted interviews at two clinics (C14 and C08), 6 months after each clinic's transition into the intervention phase. Data collection occurred between January and April 2024. Trained research assistants, (JK—male and JA—female), conducted IDIs with people living with HIV and hypertension. The PLHIV client interviews were conducted in Luganda, a widely spoken language in the Kampala and Wakiso districts. Additionally, FA (female), a social scientist with extensive expertise in the subject matter, conducted KIIs with HCPs. The HCP interviews were conducted in English. Each interview lasted approximately 30−45 minutes. Two interview guides were used: one for IDIs, and another for KIIs. The questions explored issues such as how easy or challenging it was to integrate hypertension care into the existing DSD models, clients’ experiences in receiving hypertension care, barriers or challenges they encountered and how beneficial they found the integration of hypertension care into these models.

### 2.5 Data analysis

#### 2.5.1 Quantitative analysis

We conducted univariate analyses to describe the socio‐demographic characteristics of the cohort. We summarised continuous variables using medians and interquartile ranges and presented categorical variables as percentages and frequencies.

A generalised estimation equation (GEE) model was used to account for repeated measures within participants over time. The binary outcome variable was enrolment in intensive DSD models (FBIM and FBG) versus the other DSDs. A logit link function was used, with clinic (categorised: 1–8), time from start of intervention (continuous) and the clinic*time interaction term as predictors. Clinic C12, the first to implement the intervention and the clinic with the longest follow‐up duration, served as the reference. An exchangeable correlation structure was assumed, and robust standard errors were used. Clusters were defined by participant ID where the repeated measures were assumed to be correlated, with 3164 clients making 6575 visits (range 1–11) with a mean of two observations. Comparisons were made with the exchangeable correlation structure which showed consistent results. Wald tests were used to establish overall clinic effects and those of each clinic*time interaction. Clinic‐level margin plots were made to check the consistency of predictive margins for being in intensive DSDs. Analyses were performed in Stata 18.5 and *p* < 0.05 was considered statistically significant.

#### 2.5.2 Qualitative analysis

All interviews were audio recorded and transcribed verbatim by an external consultant. The updated CFIR guided our qualitative data analysis. Two social scientists (FA and CK) reviewed the 14 transcripts and conducted rapid thematic analysis, manually coding the transcripts to identify preliminary codes which were then synthesised into emerging themes related to integrating hypertension care within DSD models. We held team meetings (ARK, FA, CK, JBK and FCS) to discuss the codes and emerging themes, and resolve any disagreements until a final codebook was agreed upon. This analysis examined PLHIV's and HCP's experiences with the integration of hypertension care within DSD models. We present the results of this analysis using the CFIR domains and constructs [26], along with themes and illustrative quotes from the participants.

### 2.6 Conceptual framework

We utilised the updated CFIR to identify key domains influencing implementation [26]—we sought to identify domains and constructs that supported or hindered the integration of hypertension care in DSD models. We identified the updated CFIR constructs (innovation domain and inner setting) as being relevant to understanding how the innovation was received and delivered by PLHIV and HCPs, respectively. We were interested in understanding how the intervention was beneficial to both PLHIV and HCPs, whether it was adaptable or not with existing DSD models and how easy or difficult it was to integrate hypertension care into DSD models of care. Following our analysis, we mapped our themes onto the identified CFIR domains and constructs.

### 2.7 Data integration

We utilised the narrative approach in which qualitative findings provided a more in‐depth understanding and context to the quantitative results, even though they are presented separately in the results [29].

### 2.8 Ethics approval and consent to participate

The Makerere University School of Medicine Research and Ethics Committee (Mak‐SOMREC‐2022‐40) and the Uganda National Council for Science and Technology (HS2398ES) approved the study. We also obtained administrative clearance from the leadership of the Uganda Ministry of Health, Kampala and Wakiso districts. We received a waiver of consent to collect routine clinical data while participants provided informed consent to participate in qualitative interviews.

## 3 RESULTS

### 3.1 Quantitative results

#### 3.1.1 Participant characteristics

**Figure 1:**
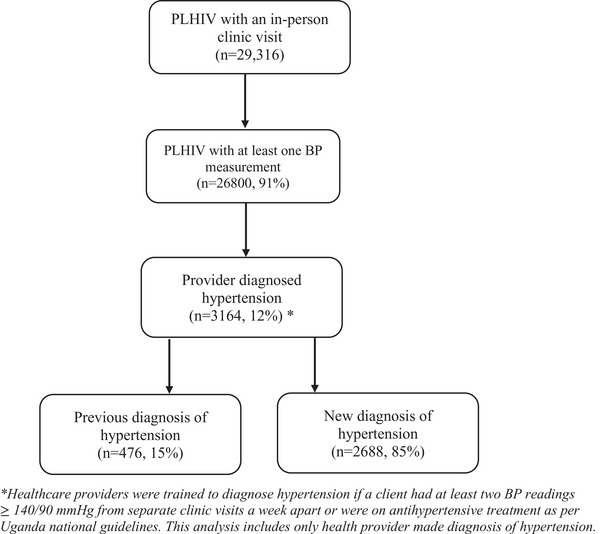
**Diagram showing hypertension screening and diagnosis cascades**. Abbreviations: BP, blood pressure; PLHIV, people living with HIV.

Overall, across the eight “Hypertension PLUS” clinics, 29,316 sought HIV care from the clinics. Of whom, 26,800 (91%) had at least one BP measurement, and 3164 (12%) PLHIV had hypertension; 2688 (85%) of whom were diagnosed during the intervention, which included higher rates of BP screening with newly available and functioning BP cuffs. Over two‐thirds of the participants were females with a median age of 44 years (IQR; 38–52). The participants were predominantly getting care from urban public clinics in Kampala (Table 1 and Figure 1).

**Table 1:** Characteristics of PLHIV diagnosed with hypertension

	Previously diagnosed	Newly diagnosed	All
Variable	*n* = 476	*n* = 2688	*N* = 3164
Age (years)	44 (38−52)	47 (39−54)	46 (38−54)
Age category			
18−35 years	92 (19.3)	460 (17.1)	552 (17.4)
36−49 years	228 (47.9)	1123 (41.8)	1351 (42.7)
50+ years	156 (32.8)	1105 (41.1)	1261 (39.9)
Sex			
Female	326 (68.5)	1842 (68.5)	2168 (68.5)
Male	150 (31.5)	846 (31.5)	996 (31.5)
District			
Kampala	417 (87.6)	2131 (79.3)	2548 (80.5)
Wakiso	59 (12.4)	557 (20.7)	616 (19.5)
Clinic type			
Private not for profit	62 (13.0)	313 (11.6)	375 (11.9)
Public	414 (87.0)	2375 (88.4)	2789 (88.1)

*Note*: Data expressed as median (interquartile range) for continuous variables and number (%) for categorical variables.

Abbreviation: PLHIV, people living with HIV.

#### 3.1.2 Trends in DSD models for PLHIV and hypertension following implementation of hypertension PLUS

There was considerable heterogeneity in DSD model use across the eight sites. Except for C10, all other clinics had significantly lower odds of using the intensive models (*p* < 0.001) relative to the reference clinic. The Wald test revealed a significant overall clinic effect (χ^2^ (7) = 1276.82, *p* < 0.001). The time from the start of the intervention also showed a statistically significant main effect (OR 1.127 [95% CI 1.059−1.199], *p* < 0.001), indicating that uptake increased significantly over time across all clinics.

**Figure 2:**
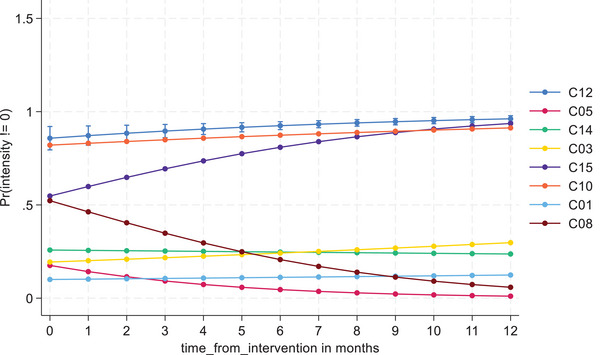
**Model predicted clinic‐level trends of differentiated service delivery (DSD) enrolment into intensive DSD models over time**.

Most clinics had relatively stable use of more versus less intensive DSD models over time; however, some clinics saw considerable changes (Supporting information File S5). Overall, the Wald test showed the interaction between clinic and time was statistically significant (χ^2^ (7) = 69.94, *p* < 0.001). The predictive model plot shows that some clinics favoured intensive models for PLHIV with hypertension, while others kept these participants in less intensive models. Both clinics that contributed qualitative data to this analysis (C08 and C14) showed statistically significant clinic*time interactions (*p* < 0.001), demonstrating a shift towards less intensive models over time after implementation. One clinic—C15—showed a non‐statistically significant (*p* = 0.827) trend towards intensive model use over time, possibly an artifact of the short 2‐month period of observation at this clinic since it was the last site to implement hypertension PLUS (Table 2 and Figure 2).

**Table 2:** Odds of enrolment into intensive DSD models compared to the reference clinic (C12; representing the first clinic to rollout the intervention)

More intensive DSDs	OR	Robust SE	*p*
Clinic			
C12	1.000		
C05	0.035 (0.015−0.084)	0.016	*p* < 0.001
C14	0.058 (0.033−0.100	0.016	*p* < 0.001
C03	0.040 (0.020−0.080)	0.014	*p* < 0.001
C15	0.201 (0.092−0.438)	0.080	*p* < 0.001
C10	0.759 (0.431−1.338)	0.220	0.341
C01	0.018 (005−0.072)	0.013	*p* < 0.001
C08	0.182 (0.096−0.345)	0.060	*p* < 0.001
Time from intervention start	1.127 (1.059−1.199)	0.036	*p* < 0.001

Abbreviations: DSD, differentiated service delivery; OR, odds ratio; SE, standard error.

### 3.2 Qualitative results

#### 3.2.1 Participant characteristics

Six HCPs participated in the KIIs, half of whom were males with a median age of 34 years (IQR: 27–40). Of the eight PLHIV with hypertension who were interviewed, 50% were males and the median age was 56 (IQR: 52–61) (Table 3).

**Table 3:** Characteristics of participants who participated in the qualitative interviews

	Healthcare providers	PLHIV with hypertension
	*n* = 6	*n* = 8
Age	34 (27, 40)	56 (52−60.5)
Sex		
Male	3 (50)	4 (50)
Female	3 (50)	4 (50)
Cadre		
Medical doctor	2 (33.3)	–
Nurse	2 (33.3)	–
Clinical officer	1 (16.7)	–
Pharmacy technician	1 (16.7)	–
Years of practice	8.5 (2−16)	−
Hypertension status		
Newly diagnosed	−	4 (50)
Previously diagnosed	−	4 (50)

*Note*: Data expressed as median (interquartile range) for continuous variables and number (%) for categorical variables.

Abbreviation: PLHIV, people living with HIV.

The following CFIR domains were dominant determinants of hypertension care implementation into existing DSD models. Some were barriers (−), while others were facilitators (+) (Table 4).

**Table 4:** Summary of key themes, interview questions and quotes on integration of hypertension in DSD models

CFIR domain/construct	Interview questions	Qualitative data
**Innovation relative advantage (+)** *Integration of hypertension into differentiated service delivery (DSD) models addresses clients’ needs, reduces frequency of visits to clinic, transportation costs, improves medication adherence*	**PLHIV and h ealthcare providers** What have been the benefits of the intervention? **PLHIV** If you were given a choice to receive hypertension services separately from HIV services or combined services at the same time and with the same provider, which option would you choose and why?	*“I choose to get the integrated service because if you say you get hypertension and HIV services on separate days you will get inconvenienced. Like me who lives faraway I will have to travel two times as compared to integrated services where you come once and get all your medicines at once”* (Female, 50 years, Community drug distribution point, C08)
**Innovation adaptability (+)** *Integration of HIV hypertension in facility models is possible due to the availability of hypertension medicines* *Possible to adapt to existing models of care and may not disrupt services for PLHIV* *Synchronisation of HIV hypertension refills is possible for PLHIV receiving care through fast track drug refill*	**Healthcare providers** How easy or difficult has it been to integrate hypertension care into HIV DSD models? What changes have you made within the clinic to accommodate implementation of hypertension‐HIV integration?	*“If they [PLHIV] have controlled blood pressure, they qualify for fast track drug refill (FTDR) so they will get three months of ART and even the anti‐hypertensives [medications] they will get three months [refills] because the stock [hypertensive medication] is there. If they [PLHIV] have uncontrolled BP, they're returned to the facility based individual management where they have to be seen by a clinician”* (Medical officer, C14) *“When staff go for community outreach, they carry their drugs with the dispensing log. The dispenser who is part of the team is tasked with recording whatever has been done during the community outreach”* (Pharmacy staff, C14)
**Innovation design (+)** *Positive perception of integration of hypertension care into DSD models, it encourages client centred care, ensures holistic care, de‐fragmentation of services, comprehensive care, more effective client management, alignment of hypertension/HIV services*	**Healthcare providers** What are your thoughts about integrating hypertension care within HIV services? How well is the clinic integrating hypertension into HIV services? **PLHIV** Tell me about your experience receiving both HIV and hypertension services at this facility under the new approach where services are combined.	*“According to the new guidelines, we were told to give them [PLHIV with hypertension] their [multi] month [refills] and they [PLHIV] just come back here for refills and medication because when they come back we take their blood pressure and refill their medication; initially we were not supposed to give them those refills for hypertension medications, but now with the new guidelines as of 2022 we give them [multi month dispensing] for hypertension”* (Nurse, C14) *“I just want to get the combined services because traveling tires me out; if you come one month and get the antiretroviral therapy and come back another month to get a blood pressure medication that is an inconvenience for me to travel. With [integrated services] it's easier for me because I come at one and I don't have to worry about coming back”*. (74‐year‐old Male, C08)
**Innovation complexity** (−) *Negative perception if integrating hypertension services in some of the DSD models like retail pharmacy model which are run privately, misalignment of clinic visits*	**Healthcare providers** What challenges or barriers have you experienced receiving or delivering hypertension and HIV services? **PLHIV** What, if any are the challenges you may have encountered while accessing HIV and hypertension services in a combined manner at this facility	*“If a client is diagnosed with hypertension during a previous visit, the best option is to take them off the FTDR point. But, for those diagnosed with hypertension at the FTDR table after missing an earlier appointment, it becomes inconvenient—they have to go back to the clinic and start regular visits again. …it involves a long line.…….”* (Medical officer, C14) *“Some of us receive services from the community clinic so when we come here to see the doctor but the blood pressure medicine is not available in the community—so I would ask that in the community too we should be able to get the BP medicine”* (PLHIV, C14)

CFIR inner setting domain		
**Available resources, materials and equipment (+)** *Availability of hypertension treatment protocol, functional blood pressure devices and anti‐hypertensive medicines have supported intervention*	**Healthcare providers** What kind of support, materials have facilitated integration? How adequate has the support been to integrate hypertension services into differentiated service delivery models? **PLHIV** What kind of support have you received from staff at this facility on managing both HIV and hypertension conditions?	*‘‘What I would say has facilitated integration, the additional blood pressure machines, the trainings, and then the drugs, those have helped us with the integration* (Nurse, C14) *They [health care providers] ask about how I feel, they come and health educate us here and tell us how to behave when taking medicine and taking it on time; the health care provider comes and teaches us. When you ask questions, they are answered well. I don't see anything missing because if I've got my antiretroviral therapy, and I've got my blood pressure medication then what more am I asking for?* (74‐year‐old Male, C08)
**Access to knowledge and information (+)** *Availability of training and health education on hypertension management positively impacts the intervention*	**Healthcare providers** How has the training and support received impacted or influenced your practice of providing integrated hypertension and HIV services?	*“Training in hypertension management has influenced us, given us more knowledge of how to handle hypertension cases in PLHIV”* (Clinical officer, C08)

Abbreviations: CFIR, consolidated framework for implementation research; DSD, differentiated service delivery; FTDR, fast track drug refill; HIV, human immunodeficiency virus; MMD, multi‐month dispensing; PLHIV, people living with HIV.

#### 3.2.2 **Innovation adaptability** (degree to which intervention can be adapted, tailored, refined, reinvented to meet local needs)

##### 3.2.2.1 Fast track drug refill model adaptable to integration (±)

HCPs generally expressed that the integration of hypertension in DSD models was easily adaptable. They highlighted that models such as the fast track drug refill were particularly amenable to adaptation, especially for PLHIV who had controlled BP and suppressed viral load (Supporting information File S6). PLHIV with controlled BP continued to receive both HIV and hypertension care in this model, as illustrated in the following excerpt:

“*If they [PLHIV] have controlled BP, they qualify for fast track drug refill so they will get three months of ART and even the anti‐hypertensives [medications] they will get three months [refills] because the stock [hypertensive medication] is there*…” (Medical officer, C14)

HCPs re‐organised client flow at the fast track drug refill point to incorporate routine BP measurement and triage. As described in the excerpt below, PLHIV with uncontrolled hypertension were reassigned to FBIM for more comprehensive care, while those who had controlled hypertension continued to receive hypertension medication refills through this model.

“*Fast track drug refill clients did not want to be removed from their models. They are used to getting six months of ART refills now you want to bring them down [routine clinic] and give few months, they were not happy but we try, we counsel and explain. And now with a BP machine at the FTDR desk at least we can still measure them before we give them a refill*…” (Nurse, C14)

##### 3.2.2.2 Reassignment across DSD models (−)

In the early stages of implementation of the integrated care, HCPs tended to reassign PLHIV with uncontrolled BPs from a less intensive model such as fast track drug refill to an intensive model like FBIM.

HCPs also noted that within facility‐based less intensive DSD models, clients valued the convenience of receiving 6‐month ART refills and expressed a desire for a similar approach to anti‐hypertensive medication refills. Challenges were noted, mainly when clients were newly diagnosed with hypertension as elaborated by a medical officer in the excerpt below:

“*If a client is diagnosed with hypertension during a previous visit, the best option is to take them off the fast track drug refill point. But, for those diagnosed with hypertension at the fast track drug refill point after missing an earlier appointment, it becomes inconvenient—they have to go back to the clinic and start regular visits again. …it involves a long line*.…….” (Medical officer, C14)

##### 3.2.2.3 Adaptation of integration in CDDP models (±)

HCPs reported that integrating hypertension care into outreach HCP‐led CDDP models was straightforward. To seamlessly integrate hypertension within this model, HCPs ensured that they carried supplies (BP devices, medications, treatment algorithm and dispensing logs) needed for the provision of hypertension care. HCPs sought to align hypertension and ART services. HCPs tried not to disrupt ART services by providing additional counselling and MMD for hypertension.

“*Usually, they [community drug distribution point‐outreach team] also have an algorithm for hypertension treatment; those who go for outreach, we give them an [hypertension treatment] algorithm. They have a dispensing log. They go with [ART and antihypertensive] medications. They have to make sure that they have to check the appointment book and see those ones who need [hypertension medications] refill on different appointments. So, they can know, they have to make sure that they go with hypertension medications because they have clients there*.” (Medical officer, C08)

#### 3.2.3 **Innovation relative advantage** (the innovation is better than other available innovations or current practice)

##### 3.2.3.1 Perceived benefits of integration of hypertension in DSD models (±)

PLHIV emphasised that receiving treatment and medication for both HIV and hypertension during a single visit was extremely beneficial, convenient and reduced the need for multiple clinic visits. This addressed the challenge of seeking care at multiple locations, as illustrated in the excerpt below.

“*I choose to get the integrated service because if you say you get for hypertension and HIV on separate days you will get inconvenienced like me who stays very far it means you will have to travel two times as compared to the integrated service where you will have to come once and you go back with all the medications at once*.” (female 50 years, Community drug distribution point, CO8)

#### 3.2.4 **Innovation complexity** (the innovation is complicated, which may be reflected by its scope and/or the nature and number of connections and steps)

##### 3.2.4.1 Lack of integration in the CRPDDP model (−)

Integrating hypertension care into this model was not possible because community retail private pharmacies lacked free hypertension services. Interviews with PLHIV revealed that community retail pharmacies lacked hypertension services and they expressed the need for BP devices and medications to facilitate the provision of hypertension care within this model. HCPs also noted the lack of integrated HIV and hypertension services and often advised PLHIV with hypertension in this model to return to the clinic to receive hypertension care, as illustrated by the following HCP's account:

“*Clients in the community retail pharmacy drug distribution point model are not screened for hypertension. The government is still working on that. For those already on hypertension treatment, they always come back to the facility… they come back here, we rescreen them, and then provide the refill*.”

Interviewer (I): *“So, in the community, they only receive the ART?”*

Respondent (R): *“Yes, they only receive ART.”* (Nurse, C14)

Participants were often advised to purchase the medications from nearby pharmacies if they preferred not to return to the clinics, as described in the excerpt below.

“*For the community retail pharmacy drug distribution point, many of them [PLHIV] who were known [to have] hypertension were buying their own [anti‐hypertensive] medications, they would not come to the HIV clinic for hypertension services but now some of them are coming to the clinic for hypertension only and then [get] ART refills at the community retail pharmacy drug distribution point*.” (Nurse, C14)

#### 3.2.5 **Innovation design** (innovation is well designed and packaged including how it is assembled, bundled and presented)

##### 3.2.5.1 Integration consistent with the Ministry of Health (MoH) guidelines (±)

HCPs explained that integrating hypertension care into the DSD models involved aligning hypertension and HIV care. This approach aligns with the MoH guidelines, which recommend providing MMD for both HIV and hypertension medications to clients with controlled viral load and BP.

“*According to the new guidelines, we were told to give them [PLHIV with hypertension] their [multi] month [refills] and they [PLHIV] just come back here for refills and medication because when they come back we take their BP and refill their medication; initially we were not supposed to give them those refills for hypertension medications, but now with the new guidelines as of 2022 we give them [MMD] for hypertension*.” (Nurse, H1401)

##### 3.2.5.2 Appreciation of the synchronisation of services (±)

PLHIV appreciated the alignment of hypertension and HIV visits, which enabled them to receive synchronised HIV and hypertension care and reduced the need for disjointed clinic visits and associated travel expenses as described below.

“*What I benefit from this, is being helped because when I come on the day I was told to come back, it is all done at once because that up and about movement inconveniences me in terms of transportation …if they give me about a month, when I come back after that period they check my BP or if it is three months, they do it all after those months but they don't tell me to either wait three days or after a week and I come back, no; if they give me one month to come back to give me the antiretroviral therapy, even the BP medication shall be for one month*.” (PLHIV, 68 Male, Community drug distribution point, C08)

#### 3.2.6 **Available resources** (resources are available to implement and deliver the innovation)

##### 3.2.6.1 Availability of BP devices to integrate hypertension care in community outreaches (+)

HCPs reported that the availability of BP devices and medications were facilitators for the integration of hypertension care into existing DSD models.

“*What I would say has facilitated integration, the additional BP machines, the trainings, and then the medicines, those have helped us with the integration*.” (Nurse, C14)

#### 3.2.7 **Access to knowledge and information** (guidance/training is accessible to implement and deliver the innovation)

##### 3.2.7.1 Training on hypertension care facilitated its integration in DSD models (±)

HCPs reported training on hypertension was greatly beneficial and improved their confidence to manage hypertension. They explained that training in hypertension management expanded their knowledge and competency to diagnose, monitor and treat PLHIV with hypertension.

“*The trainings have helped very much‐for me as a clinician it added to my knowledge on how to prescribe because now, I can confidently prescribe, I know what I am doing because of the training we received*.” (Nurse, C14)

## 4 DISCUSSION

This study assessed the effect of integrating hypertension care into HIV care on the enrolment of PLHIV with hypertension in various DSD models, using interrupted time series analysis. We also explored the experiences of HCPs in delivering, and PLHIV with hypertension in receiving, hypertension care within these models using the updated CFIR. Our findings showed an increased likelihood of enrolment of PLHIV with hypertension into intensive DSD models, especially in the initial phases of the study (2−16 months); however, there was significant variation across the clinics with two clinics demonstrating a statistically significant trend towards less intensive models over time. Qualitative findings revealed that integration of hypertension care into HCP‐managed less intensive models was feasible, beneficial, adaptable and enhanced person‐centred care. To integrate HIV hypertension care in DSD models, HCPs temporarily transitioned clients into intensive models until the desired clinical status was achieved. Although clients initially resisted the transition, those who achieved BP control were eventually reassigned back to less intensive models during the course of the intervention. Integrating hypertension care into the community retail pharmacy model proved challenging, resulting in fragmented HIV and hypertension care.

As opposed to client‐led versions of less intensive models (e.g. CRPDDP), less intensive models managed by HCPs (e.g. FTDR and CDDP‐outreach) were easily adapted for hypertension care integration. In contrast, integrating hypertension services into the less intensive client‐led models was limited, resulting in fragmented care for hypertension and HIV. The integration of hypertension care into a less intensive model was largely attributed to HCP‐initiated modifications. These included implementing BP measurement and triage at fast track drug refill points, as well as carrying anti‐hypertensive medications, BP devices and treatment algorithms to community outreach points. Clients receiving care through the community retail pharmacy model were often encouraged to purchase their medications or receive fragmented hypertension medication refills from pharmacy points.

Transitioning clients with uncontrolled hypertension into intensive facility‐based models such as FBIM and FBGs was a common strategy for ensuring clients received medically appropriate care. However, qualitative findings revealed that some clients initially resisted transition and opted to receive disjointed care. With time, clients were transitioned back to less intensive models as they achieved controlled BP, or good adherence to anti‐hypertensive medication.

The use of the CFIR enabled the identification of key factors influencing the integration of hypertension care into DSD models. Specifically, the interplay between innovation, access to knowledge and availability of resources emerged as critical determinants of success. At the two clinics (C08 and C14) showing the greatest adaptability over time, the integration of hypertension care into HCP‐managed less intensive models, such as FTDR and CDDP‐outreach, was possible due to the availability of resources (e.g. medications and BP devices) and access to hypertension training for HCPs. HCPs implemented several innovations to support integration. These included conducting BP monitoring at fast track drug refill points, identifying clients with uncontrolled hypertension and redirecting them to clinics for enhanced management. HCPs also transported BP machines, medications and treatment algorithms to community settings, ensuring the delivery of integrated HIV and hypertension care directly to clients. However, despite the availability of medications and BP devices at clinics, HCPs viewed it as not feasible to extend these services to the small number of clients receiving HIV medication refills from community retail pharmacies. This limitation resulted in fragmented care for these clients with comorbid hypertension, highlighting a significant challenge to achieving comprehensive integration.

Consistent with findings from other studies [17, 30], availability of resources (BP devices and medications), training of HIV HCPs, task shifting and use of simplified protocols enhanced the integration of hypertension care into HIV services, as evidenced by the markedly high hypertension screening and detection rates. However, the high prevalence of hypertension presents new and unique demands for the integration of hypertension care into an already mature HIV service delivery programme, potentially necessitating adjustments to client flow and re‐allocation to various DSD models. For example, PLHIV with uncontrolled hypertension might require a transition to an intensive model, while clients who have achieved control of both HIV and hypertension may be transitioned to less intensive models. The national guidelines need to be more explicit on the eligibility for transition from one model to another based on HIV and hypertension control status as well as the clients’ preferences.

The CRPDDP model was described as less adaptable for hypertension services. Operationalising this model by ensuring consistent availability of resources (BP medications and devices) and training of the community pharmacy staff are needed to integrate hypertension care into the CRPDDP model, but require considerable resources, planning, and alignment of public health and business incentives. The benefits of receiving pharmacy‐based hypertension services such as adherence counselling, BP monitoring and optimising hypertension management have been documented in the African region [31]. To increase the client‐centeredness of integrated care, hypertension care for PLHIV should be expanded to pharmacy‐based models. Furthermore, insights from South Africa's Central Chronic Medicine Dispensing and Distribution programme could be modelled into Uganda's CRPDDP and expanded to provide hypertension medication refills, BP monitoring, counselling on side effects not only to PLHIV with hypertension but also to individuals with NCDs [32].

One of our study's strengths is the mixed‐methods design, which revealed the nuances in perceptions in receiving and experiences of delivering and receiving integrated HIV hypertension care across the intensive and less intensive DSD models. Second, the study applied selected CFIR constructs to explore how integrated care was received by PLHIV and delivered by HCP. Third, by interviewing both HCPs and PLHIV, the study identified demand‐side and supply‐side challenges affecting the integration of hypertension care within DSD models. Finally, the GEE model illustrated changes over time in the pooled sample as well as by individual clinic.

One of the limitations of this paper is it does not address the effect of integration on clinical outcomes, such as BP control and so on, so as not to interfere with reporting of the primary results of the clinical trial. In future analyses, we will aim to evaluate the effect of providing hypertension care in various DSD models on these clinical outcomes. The study provided free medications and BP devices and implemented a human resource‐intensive strategy. However, the scope of this paper does not include an assessment of the cost‐effectiveness of integrating hypertension care into DSD models. A cost‐effectiveness analysis is planned after trial completion to provide further insights into the financial feasibility of this approach. Due to time limitations, the qualitative data was obtained from two clinics. This may have resulted in missing insights from other clinics, particularly those with a higher proportion of clients enrolled in intensive models of care. Lastly, for brevity, we focused on two CFIR domains—Innovation and Inner setting—to explain the most important barriers and facilitators of care emerging from our qualitative data; however, other CFIR domains could be considered in future work.

## 5 CONCLUSIONS

Integrating hypertension care into DSD models is feasible and adaptable in peri‐urban and urban settings in Uganda. However, it requires transitioning PLHIV between models based on clinical needs. This approach enhances person‐centred care and allows for more comprehensive management of hypertension; however, careful planning is needed to address challenges related to resource availability, client preferences and staff training. There is a need to facilitate client transition with clear education. This approach will enhance client education on the importance of transitioning to intensive DSD models when needed for better hypertension management, which will mitigate resistance and improve adherence to care protocols. Additionally, strengthening community DSD models through the provision of adequate resources (medications, BP devices and tools) and training of non‐physician HCPs is essential for the delivery of integrated HIV hypertension care in Ugandan settings.

## COMPETING INTERESTS

The authors have no competing interests to declare.

## AUTHORS’ CONTRIBUTIONS

FCS, CTL, ARK, JIS, MM, IS, JK and DS designed the research study and secured the funding for this research. JBK, FCS, CTL and ARK conceptualised the manuscript. JBK drafted the manuscript. ENM, FA, CFL and CK participated in data collection. FA, CK, ENM and CFL analysed qualitative results. LG analysed quantitative data and FA, CK, ENM and CFL analysed qualitative results. ARK, FCS and CTL provided a detailed review of the initial drafts. All authors reviewed and contributed to the final manuscript.

## FUNDING

The research presented in this publication was funded by the National Heart, Lung, and Blood Institute (NHLBI) of the National Institutes of Health (USA) through Award number 1UG3HL154501(awarded to authors FS and CTL). The content is the sole responsibility of the authors and does not necessarily reflect the official views of the National Institutes of Health. The funders had no role in the study design, data collection, analysis, interpretation, manuscript writing, or the decision to submit the work for publication.

## DISCLAIMER

The content is solely the responsibility of the authors and does not necessarily represent the official views of the National Heart, Lung, and Blood Institute, the National Institutes of Health or the U.S. Department of Health and Human Services.

## Supporting information




**Supplemental file 1**: PULESA‐Uganda study intervention components.
**Supplemental file 2**: Characteristics of HIV clinics participating in the Hypertension PLUS arm of the PULESA‐Uganda study.
**Supplemental file 3**: Differentiated service delivery models in Uganda.
**Supplemental file 4**: Stepped wedge cluster randomised trial design schematic.
**Supplemental file 5**: Staked bar graphs showing the distribution of study visits by DSD model over time at the eight Hypertension PLUS clinic sites.
**Supplemental file 6**: Comparing PLHIV level versus provider‐level themes.

## Data Availability

The datasets utilised in this study can be obtained from the corresponding author upon reasonable request.
