# Supplementary material for: Early findings from the integration of hypertension care into differentiated service delivery models for HIV in Uganda: a mixed‐method study
Source: J Int AIDS Soc. 2025 Jul 7;28(Suppl 3):e26499. doi: 10.1002/jia2.26499 (PMC12232483; doi:10.1002/jia2.26499)
Supplement: Supplementary file 1 — Supplemental file 1: PULESA‐Uganda study intervention components. Supplemental file 2: Characteristics of HIV clinics participating in the Hypertension PLUS arm of the PULESA‐Uganda study. Supplemental file 3: Differentiated service delivery models in Uganda. Supplemental file 4: Stepped wedge cluster randomised trial design schematic. Supplemental file 5: Staked bar graphs showing the distribution of study visits by DSD model over time at the eight Hypertension PLUS clinic sites. Supplemental file 6: Comparing PLHIV level versus provider‐level themes. [file JIA2-28-e26499-s001.pdf]

SUPPLEMENT MATERIALS

Supplemental file 1: PULESA study intervention components

HYPERTENSION BASIC	HYPERTENSION PLUS (All components of HYPERTENSION BASIC plus)
1. Access to evidence based anti-hypertension medications (Amlodipine 10mg, Valsartan 180mg and hydrochlorothiazide25mg)<br><br>**a.** Treatment algorithm to aid prescription of the above-mentioned medications.	4. Comprehensive training on hypertension provided to HCP through<br><br>a. One on one coaching with clinic staff<br>b. Online case discussions and<br>c. Periodic refresher trainings on hypertension based on need
2. Access Blood Pressure devices<br>**a.** Two or more Welch Allyn Pro2000 BP devices were distributed to study clinics<br>**b.** Health care workers trained on BP measured using digital devices	5. Adoption of Differentiated Service Delivery (DSD) for HYPERTENSION care<br>a. Adoption of multi month dispensing of hypertensive medications to eligible PLHIV with HYPERTENSION<br>b. Incorporation of Hypertension care in community-based drug distribution model<br>c. Aligning HIV and HYPERTENSION visits
3. Baseline basic training of HCP on hypertension.<br>**a.** Trainings on hypertension conducted by study medical doctor and nurses at the start of run-in period<br><br>**b.** The training covered topics like BP measurement, HYPERTENSION diagnosis, treatment of hypertension using a standardized algorithm and	6. Remote patient monitoring<br><br>a. Clinic staff provided community BP monitoring cards to document community BP measurements.
	7. Quarterly performance feedback on hypertension care metrics.<br>a. Quarterly performance reports shared and discussed with the clinic teams.<br>b. Reports included data on HYPERTENSION care [illegible]

Supplemental file 2: Characteristics of the HIV clinics participating in the Hypertension PLUS arm of the PULESA study

Clinic	Location	Type	No. Clients†	DSD Models	Duration
C12	Kampala	Public	1331	FBIM, FTDR	16 months
C01	Wakiso	Public	910	FBIM, FTDR	14 months
C14	Kampala	Public	8934	FBIM, FTDR, CRPDDP, CCLAD	12 months
C08	Wakiso	PNFP	1093	FBIM, FTDR, CRPDDP	10 months
C03	Wakiso	Public	859	FBIM, FTDR	8 months
C10	Kampala	Public	5700	FBIM, FTDR, FBG, CRPDDP	6 months
C05	Wakiso	Public	3129	FBIM, FTDR, CRPDDP	4 months
C15	Kampala	PNFP	8300	FBIM, FTDR, CRPDDP	2 months

†*Number of clients accessing care at the time of protocol development (April-June 2022)*

FBIM, Facility Based Individual Management; FTDR, Fast Track Drug Refill; CRPDDP, Community Retail Pharmacy Drug Distribution Point; CCLAD, Community Client Led ART Delivery

Supplemental file 3: Differentiated service delivery models in Uganda.

DSD Model	Description	Type of service (WHAT)	Location (WHERE)	Service provider (HOW)	Frequency of service (WHEN)
Facility Based Individual Management (FBIM)	Clients get a comprehensive clinical assessment on every visit to the HIV clinic.<br><br>Clients include those that have been newly initiated on ART, those with advance HIV disease and those with uncontrolled NCDs like hypertension.	Clinical assessment<br>ART refills<br>Lab tests review<br>Adherence support	PHC HIV clinic	Clinician<br>Nurse<br>Pharmacy dispenser	-1 to 2 months for newly ART initiated or complex clients.<br><br>-3 to 6 months for ART established clients.
Facility Based Groups (FBG)	Usually groups of about 15 to 40 clients that need special attention and peer support.<br><br>These may include pregnant and breastfeeding mothers, children and virally unsuppressed clients, or clients with NCDs	Psychological support (group support)<br><br>Clinical assessment<br><br>Adherence support	PHC HIV clinics	Peers<br><br>Nurse<br><br>Clinician	Depends on clients stability but groups usually meet monthly or quarterly (every 3 months).
Fast Track Drug Refills (FTDR)	ART established clients on first- and second-line ART regimen get direct refills from the dispensing point.<br><br>Clients with TB who have completed two months of intensive therapy or clients with controlled NCDs	-ART refills<br><br>-Adherence support	PHC HIV clinics	Pharmacy dispenser<br><br>Peer	3 to 6 months ART refills.
Community Drug Distribution Point	**Community Retail Pharmacy Drug Distribution Point (CRPDDP)**<br><br>Clients pick ART refills at a retail pharmacy at their convenient time<br><br>Clients visit the HIV clinic once in 12 months for labs like viral load testing and adherence assessment.	ART refills	Private pharmacies	Pharmacist/Pharmacy technician	3 to 6 months
	Community Outreach or drop in centers<br><br>HCPs to go the community to deliver HIV care to clients at specified locations.<br><br>All clients including children, adolescents and lactating mothers can receive care from the community.	ART refills<br><br>Clinical assessment<br><br>Viral load testing<br><br>Adherence support.	Community	Nurse<br><br>Clinician<br><br>Pharmacy dispenser<br><br>Peer	3 to 6 months
Community Client Led ART Delivery (CCLAD)	Established clients on ART form groups of about 3 to 6 to support each other and alternate drug pickups from the facility (HIV clinics).<br><br>Clients make annual in person visit to the HIV clinic for viral load testing.	ART refills	Community	Peer	3 to 6 months.

Supplemental file 4: **Stepped wedge cluster randomized trial design schematic**

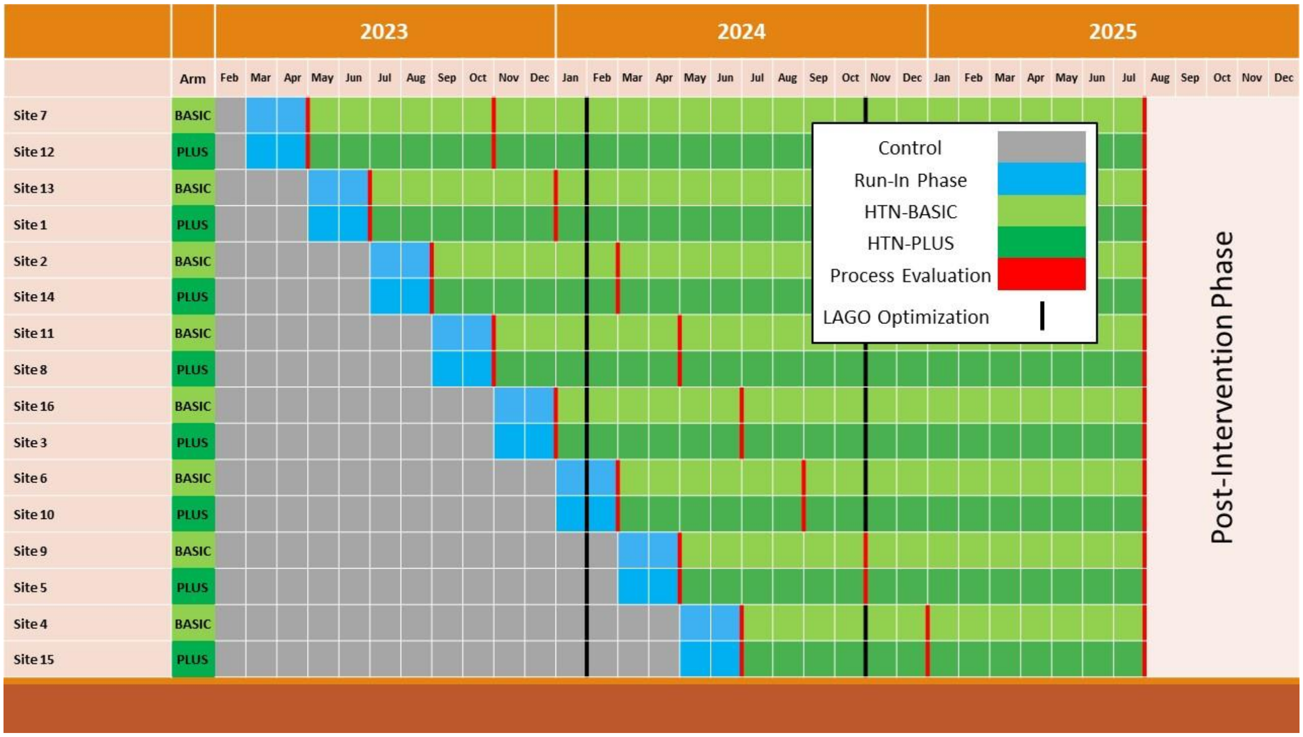

Supplemental file 5: Staked bar graphs showing the distribution of study visits by DSD model over time at the eight hypertension PLUS clinic sites.

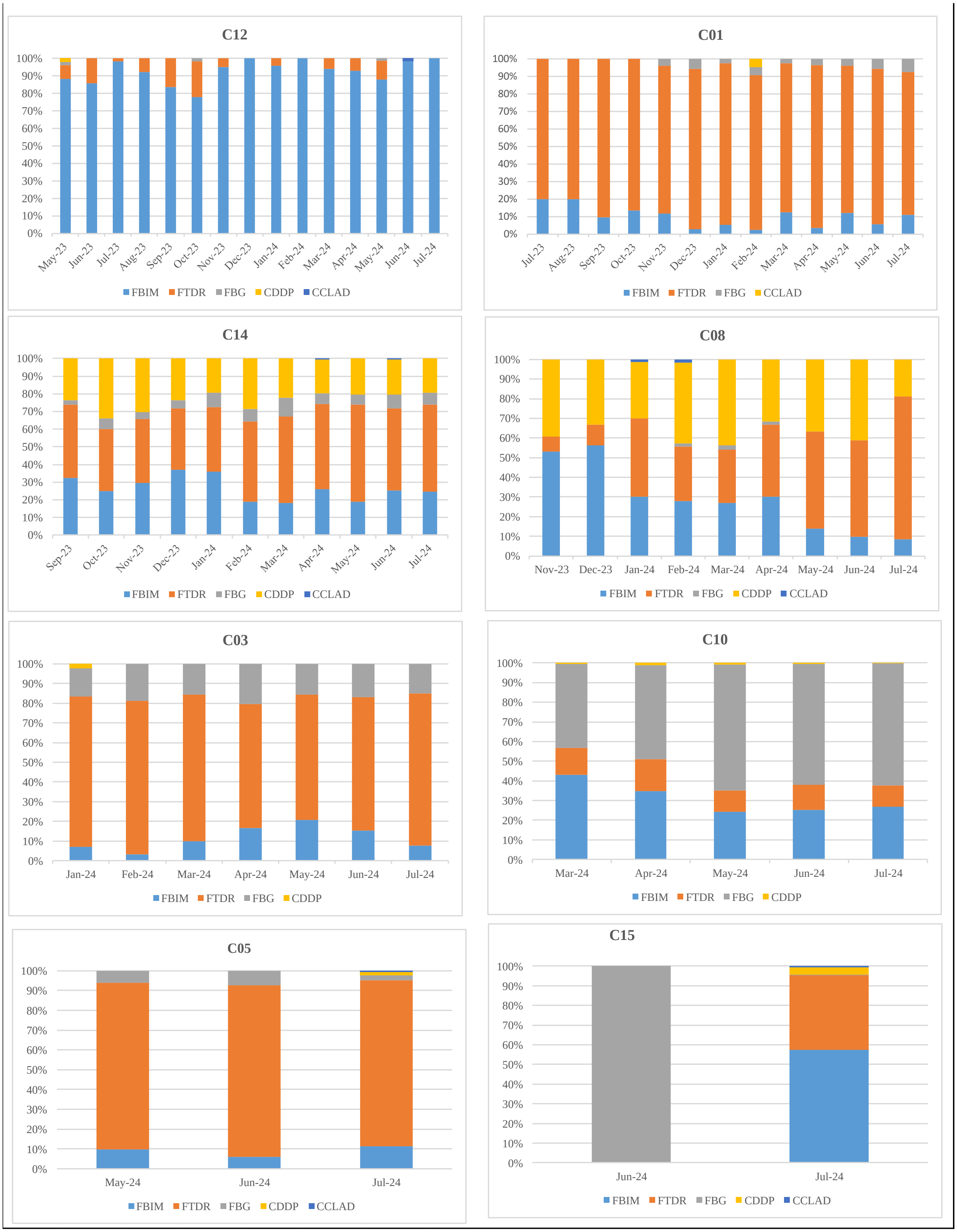

Supplemental file 6: Comparing PLHIV level versus Provider themes

PLHIV with hypertension and HCPs were asked different questions during the interviews given their specific roles as recipients of care and deliverers of care respectively. As such, although responses were different, themes were related. We only identified one difference which was under the ***inner setting*** construct of *'access to knowledge and information'* where only HCPs could address this given their exposure to training on hypertension management and integration.

**Category**	**Health care provider**	**PLHIV**
**Innovation Domain**		
Innovation adaptability *(Degree to which intervention can be adapted, tailored, refined, reinvented to meet local needs)*	▪ DSD models, FTDR easily adaptable, minimal client interaction<br>▪ Re-assignment of DSD models from less intensive to more intensive possible<br>▪ community drug distribution point – straight forward with availability of necessary resources, supplies	▪ Able to receive both hypertension/HIV care in same visit<br>▪ Alignment of refills, multi-month dispensing<br>▪ Re-assignment of DSD models with hypertension status
Innovation relative advantage *(The innovation is better than other available innovations or current practice)*	▪ PLHIV value convenience, happy to receive multi-month refills for both ART and antihypertensive medicines<br>▪ Alleviates PLHIV frequent visits for different services	▪ Receiving both during same visit beneficial vs receiving care from different service points<br>▪ Integrated care across all DSD models perceived to be more convenient, reduced need for frequent visits, care for both conditions can be received in one place
Innovation complexity *(Perceived difficulty of implementation)*	▪ Current guidelines do not adequately provide guidance/resources to integrate in pharmacy model, due to complex nature of government agreement with private pharmacies<br>▪ PLHIV in community retail pharmacy drug distribution point encouraged to seek hypertension services at facility	▪ PLHIV preference is to receive hypertension services in this DSD model, are not receptive to being sent back to facility for hypertension care<br>▪ Lack of integration for PLHIV receiving HIV care in Community retail pharmacy drug distribution point
Innovation Design *(Innovation is well designed and packaged including how it is assembled, bundled and presented)*	▪ Intervention consistent with MoH guidelines for stable clients to receive multi-month refills for both ART and antihypertensive medications	▪ Appreciation of synchronization /alignment of appointments, refills for both ART and antihypertensives
**Inner Setting**		
Available resources *(Resources are available to implement and deliver the innovation)*	▪ Sufficient resources to integrate hypertension care in DSD models i.e. blood pressure screening devices, access to consistent supply of antihypertensive medications, hypertension treatment protocol	▪ Access to hypertension services including blood pressure screening; PLHIV aware of hypertension status<br>▪ Availability of a consistent supply of antihypertensive medication at no cost

Access to knowledge and information *(Guidance/training is accessible to implement and deliver the innovation)*	▪ Access to training for HCPs on hypertension management and its integration in HIV DSD models	***No related data from PLHIV***
